# Canonical and Non-canonical Reelin Signaling

**DOI:** 10.3389/fncel.2016.00166

**Published:** 2016-06-30

**Authors:** Hans H. Bock, Petra May

**Affiliations:** Clinic of Gastroenterology and Hepatology, Heinrich-Heine-University DüsseldorfDüsseldorf, Germany

**Keywords:** neuronal development, synaptic plasticity, Alzheimer disease, amyloid precursor protein, integrin, Eph receptor, coreceptor, protein kinase

## Abstract

Reelin is a large secreted glycoprotein that is essential for correct neuronal positioning during neurodevelopment and is important for synaptic plasticity in the mature brain. Moreover, Reelin is expressed in many extraneuronal tissues; yet the roles of peripheral Reelin are largely unknown. In the brain, many of Reelin’s functions are mediated by a molecular signaling cascade that involves two lipoprotein receptors, apolipoprotein E receptor-2 (Apoer2) and very low density-lipoprotein receptor (Vldlr), the neuronal phosphoprotein Disabled-1 (Dab1), and members of the Src family of protein tyrosine kinases as crucial elements. This core signaling pathway in turn modulates the activity of adaptor proteins and downstream protein kinase cascades, many of which target the neuronal cytoskeleton. However, additional Reelin-binding receptors have been postulated or described, either as coreceptors that are essential for the activation of the “canonical” Reelin signaling cascade involving Apoer2/Vldlr and Dab1, or as receptors that activate alternative or additional signaling pathways. Here we will give an overview of canonical and alternative Reelin signaling pathways, molecular mechanisms involved, and their potential physiological roles in the context of different biological settings.

## Physiological Roles of Reelin

Reelin is best known for its role in the developing mammalian cerebral cortex, where it is secreted by Cajal-Retzius cells in the marginal zone and orchestrates the arrangement of postmitotic cortical neurons in an inside-out manner, meaning that younger neurons are located more superficially than the earlier-born neurons. This process is severely disturbed in the spontaneous mouse mutant *reeler*, where disruption of the gene encoding Reelin leads to an approximate inversion of the cortical layering. Other laminated brain structures are affected as well, resulting in a typical “*reeler* phenotype”, which includes the eponymous reeling gait as a consequence of cerebellar hypoplasia. Characteristic positioning defects of pyramidal and granule neurons in the hippocampus are found as well (reviewed e.g., by Rice and Curran, 2001; Tissir and Goffinet, 2003; D’Arcangelo, 2005). In addition to regulating layer formation in the neocortex and other laminated brain structures, Reelin functions in the developing and adult brain, where Reelin is highly expressed by GABAergic interneurons in the forebrain and by cerebellar granule neurons (Drakew et al., 1998; Pesold et al., 1998; Ramos-Moreno et al., 2006; Wierenga et al., 2010; Pohlkamp et al., 2014). Its functions include the regulation of filopodia formation, dendrite outgrowth, spine formation and synaptogenesis as well as modulation of synaptic plasticity and neurotransmitter release (reviewed by D’Arcangelo, 2005; Herz and Chen, 2006; Levenson et al., 2008; Forster et al., 2010; Levy et al., 2014).

The study of mutant mice with defects in cortical layering has significantly contributed to our current understanding of corticogenesis (Lambert de Rouvroit and Goffinet, 1998; Hatten and Heintz, 2005; Ogden et al., 2016). However, although the gene affected in *reeler* mice has been identified more than 20 years ago (D’Arcangelo et al., 1995), our knowledge of how precisely Reelin exerts its diverse functions on neuronal positioning and differentiation on a cellular and molecular level is still imperfect (Caffrey et al., 2014). In accordance with its multiple roles during different developmental stages Reelin targets different cell types, including newborn and differentiated neurons, radial glial cells, astrocytes, and possibly neural stem cells (Forster et al., 2002; Kim et al., 2002; Gong et al., 2007; Lakomá et al., 2011; Brunne et al., 2013; Brunkhorst et al., 2015). Of note, many neuropsychiatric diseases have been associated with dysregulated Reelin expression, including schizophrenia, depression, autism, temporal lobe epilepsy, and neurodegenerative disease (Impagnatiello et al., 1998; Guidotti et al., 2000; Fatemi, 2001; Persico et al., 2001; Haas et al., 2002; Sáez-Valero et al., 2003; Botella-López et al., 2006; Knuesel, 2010; Folsom and Fatemi, 2013). Reelin-responsive cells outside the central nervous system remain mostly elusive, although significant amounts of Reelin are detected in plasma and various non-neuronal tissues (Ikeda and Terashima, 1997; Smalheiser et al., 2000; Kobold et al., 2002; Lugli et al., 2003; Botella-Lopez et al., 2008), and functional effects of Reelin on blood cells such as platelets (Tseng et al., 2014), endothelial cells (Ding et al., 2016), or pancreatic cancer cell lines (Sato et al., 2006) have been described.

## Towards a Molecular Understanding of the *Reeler* Phenotype: The Core Reelin Signaling Cascade

**Figure 1:**
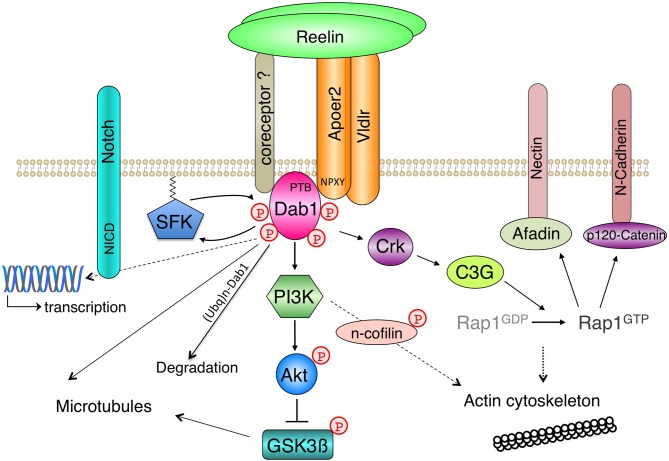
**Core Reelin signaling pathway.** A simplified scheme of Reelin-induced signaling events in neurons is shown. Based on a combination of phenotypic analysis of mouse mutants and biochemical studies a core Reelin signaling pathway was identified, where lipoprotein receptors interact with the phosphotyrosine-binding (PTB) domain of Disabled-1 (Dab1) via the NPXY motif in their intracellular domains. Oligomerized Reelin induces clustering of the receptor-Dab1 complexes, which induces tyrosine phosphorylation of Dab1 by Src family kinases (SFK) at different sites. This leads to site-specific modification of downstream signaling effectors, which is discussed in more detail in the text. The reciprocal activation of Src kinases (SFK) and Dab1, which underlies the prolonged increase of Dab1 phosphorylation after Reelin stimulation, is negatively regulated by p-Dab1 ubiquitinylation and degradation. It is unclear to what degree Apoer2 and Vldlr are coclustered by Reelin. Inhibition of the serine/threonine kinase Gsk3β via Akt was the first described lipoprotein receptor- and Dab1-dependent linear Reelin signaling cascade in neurons, which targets the cytoskeleton. Reelin binds to additional transmembrane proteins (coreceptors) that also interact with Dab1, which are not essential for the tyrosine phosphorylation of Dab1 but might be involved in receptor crosstalk and/or activation of additional downstream effectors. Long-lasting changes in responsive cells are induced by transcriptional regulation, which involves the gamma-secretase mediated release of receptor intracellular domains. The regulation of transmembrane proteins (N-Cadherin, Nectin) downstream of a Reelin-Dab1-Crk/CrkL-Rap1-dependent signaling cascade has been shown to be essential for Reelin’s role during neurodevelopment.

The discovery of spontaneous or genetically engineered mutant mouse lines that copy the *reeler* phenotype (Table 1) in combination with biochemical apporaches for identifying protein interactions proved instrumental in the discovery of a Reelin-dependent core signaling pathway (Figure 1) that underlies many of the established biological functions of Reelin in the developing and mature brain.

**Table 1:** **Mouse mutants with a *reeler*-like phenotype**.

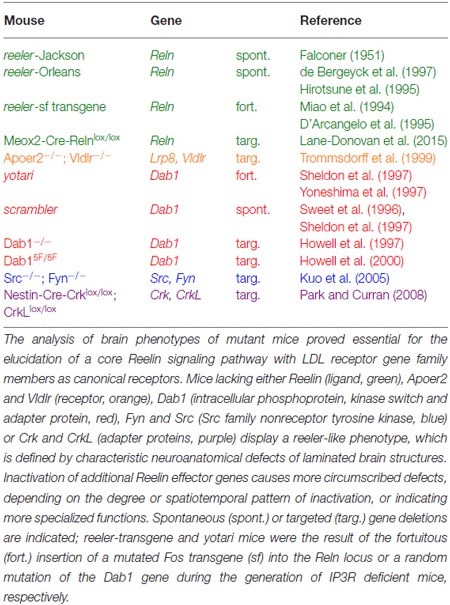

**Figure 2:**
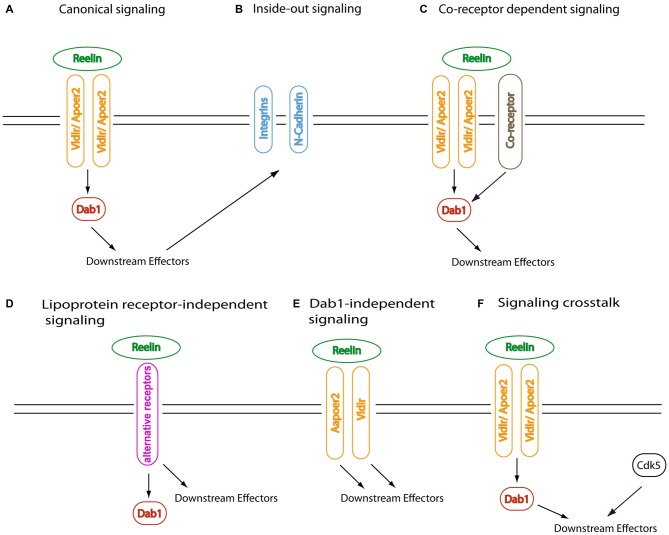
**Canoncial and non-canonical Reelin signaling.** Alternative schemes of Reelin signaling are shown. Examples for the different concepts are discussed in the text. **(A)** The canonical signaling pathway involves direct binding of Reelin via its central fragment to apolipoprotein E receptor 2 (Apoer2) and very low density lipoprotein receptor (Vldlr), which induces tyrosine phosphorylation of Dab1. This leads to the modulation of downstream signaling effectors. In this model, recruitment of SFK by Reelin-binding coreceptors is not required. **(B)** Other transmembrane receptors can be indirectly activated by Reelin-Dab1 dependent signaling in an inside-out manner; here, the receptors function as downstream effectors of the canonical Reelin signaling cascade. **(C)** In this model, the recruitment of SFK requires Reelin-binding coreceptors in addition to the canonical Reelin receptors Apoer2 and Vldlr. Cadherin-related neuronal receptors (CNR) or ephrin B proteins have been suggested to represent such putative obligatory coreceptors (see text for details). **(D)** Non-canonical activation of Dab1 signaling has been reported; this can either involve other ligands than Reelin that signal via Apoer2 or Vldlr; alternative Reelin receptors, as depicted here; or transphosphorylation of Dab1 via receptor tyrosine kinases. Reelin can also bind to alternative receptors, e.g., via its aminoterminal fragment, and induce Dab1-independent signaling cascades. **(E)** Dab1-independent Reelin signaling via Apoer2 or Vldlr has been postulated, based on phenotypic differences between the respective mouse knockout models. Apoer2-or Vldlr-specific Reelin signaling events have been reported as well, which could involve as yet unidentified receptor-specific adapter proteins. **(F)** Downstream effectors of canonical Reelin signaling function as convergence points for various upstream regulators, allowing responsive cells to fine-tune their response to external and internal stimuli in a context-dependent manner.

These include mice lacking the intracellular phosphoprotein Disabled-1 (Dab1) and compound mutant mice that lack both very low density lipoprotein receptor (Vldlr) and apolipoprotein E receptor 2 (Apoer2), which are two close relatives of the low density lipoprotein (LDL) receptor, the prototype of an endocytic receptor and founding member of the LDL receptor gene family (reviewed by Herz, 2001; Herz and Bock, 2002). This discovery is a prime example for the power of mouse genetics and came as a surprise, since so far lipoprotein receptors had not been connected with the transduction of extracellular signals via classical signaling cascades (Howell and Herz, 2001; May et al., 2003a). By demonstrating that Reelin directly binds to the extracellular domains of Apoer2 and Vldlr (D’Arcangelo et al., 1999; Hiesberger et al., 1999), which interact with the protein interaction/phosphotyrosine-binding (PTB) domain of Dab1 via the tetra-amino-acid NPXY endocytosis motif within their intracellular tails (Howell et al., 1999b; Gotthardt et al., 2000), a linear signaling pathway was established that leads to the tyrosine phosphorylation of Dab1 (Howell et al., 1999a; Rice and Curran, 1999; Figure 2A). Alternative models that were compatible with the observed mouse phenotypes (Cooper and Howell, 1999), where the lipoprotein receptors were placed downstream, or in parallel, to a Reelin-Dab1 dependent pathway, could thus be ruled out. Although either Apoer2 or Vldlr alone is sufficient for inducing Reelin-mediated Dab1 tyrosine phosphorylation in primary cultures of cortical neurons (Beffert et al., 2002; Bock and Herz, 2003), the neurodevelopmental phenotypes of the single knockout mice hint at divergent functions of both lipoprotein receptors in the transmission of the Reelin signal. These differences might be attributable to a different regional, cellular and subcellular distribution of both receptors, temporal differences in receptor expression and biochemical properties, including ligand affinities, intra- and extracelullar interaction partners, receptor turnover or processing by proteases (see below).

### Tyrosine Phosphorylation of Dab1: The Coreceptor Controversy

Subsequent studies focused on the identification of the tyrosine kinase(s) that are required for the phosphorylation of Dab1 at four tyrosyl residues close to its PTB domain, which is essential for Dab1’s function during brain development. This was demonstrated by the generation of mice that express a Dab1 protein with phenylalanine substitutions at Tyr185, 198/200, 220 and 232 (Dab1^5F/5F^; Howell et al., 2000). Since, unlike receptor tyrosine kinases, the lipoprotein receptors do not contain a cytoplasmic kinase domain, it was speculated from the beginning that a Reelin coreceptor with associated tyrosine kinase activity (either intrinsic or by forming a complex with a nonreceptor tyrosine kinase) might be required (e.g., Cooper and Howell, 1999, Figure 2C). Biochemical studies identified members of the Src family of nonreceptor tyrosine kinases as physiological Dab1 kinases (Arnaud et al., 2003b; Bock and Herz, 2003), which was confirmed genetically by the demonstration of a *reeler*-like phenotype in double-knockout mice lacking both Src and Fyn (Kuo et al., 2005). Members of the gene family of cadherin-related neuronal receptors (CNR), which are expressed in the cortical plate and bind to Fyn with their cytoplasmic domains, were reported to interact with the aminoterminal region of Reelin (Senzaki et al., 1999) and therefore considered as likely coreceptor candidates that would bring Fyn into a Reelin-lipoprotein receptor-Dab1 complex (Figure 2C). However, this interaction was not confirmed using an *in vitro* pulldown assay with recombinantly expressed CNR extracellular domain and secreted Reelin (Jossin et al., 2004), and an *in vivo* evaluation of CNR family members as obligatory coreceptors by loss-of-function studies has not been provided. In another study it was suggested that ephrin B receptors, transmembrane proteins that interact with Eph receptors to initiate bidirectional signaling (Pasquale, 2008), act as essential Reelin coreceptors, which would be required for the recruitment and activation of Src family kinases (SFK) into the lipoprotein receptor-Dab1 complex at the cell membrane (Sentürk et al., 2011; and Erratum in Nature 2011, 478:274). This model was based on both biochemical and genetic experiments, including precipitation of the *reeler* phenotype in mice carrying only one *reeler* allele on an ephrin B3 knockout background, and the analysis of ephrin B triple knockout mice, which were described to display a *reeler*-like phenotype in the neocortex, hippocampus and cerebellum (Sentürk et al., 2011). In addition, a rescue of the cortical lamination defects in organotypic cortical slice cultures from *reeler* embryos after 2 days of treatment with ephrin B ligand was reported (Sentürk et al., 2011). However, whereas binding of recombinant Reelin to the purified extracellular domain of ephrin B3 was confirmed, the clustered recombinant Ephb3 extracellular domain did not induce Dab1 tyrosine phosphorylation, as tested by Dab1 immunoprecipitation followed by immunoblotting with the phosphotyrosine antibody 4G10 (Bouché et al., 2013). To verify ephrin B transmembrane proteins as essential components of the canonical Reelin signaling cascade, the independent reproduction of a *reeler*-like phenotype in compound mutant mice would be necessary. In summary, the existence of an additional coreceptor that is required for the tyrosine phosphorylation of Dab1, which is the essential step for the intracellular propagation of the Reelin signal in the canonical pathway, has not been convincingly demonstrated to date.

On the other hand, a considerable body of evidence supports the concept that di- or oligomerization of the lipoprotein receptors Apoer2 and Vldlr, which bind to the central fragment of Reelin (Jossin et al., 2004; Yasui et al., 2010), is sufficient to explain the tyrosine phosphorylation of Dab1 induced by Reelin and its physiological consequences. Reelin is secreted as a disulfide-linked oligomer and forms higher-order multimers (Utsunomiya-Tate et al., 2000; Kubo et al., 2002; Jossin et al., 2004; Yasui et al., 2010, 2011; Manoharan et al., 2015). A conformation-dependent epitope close to the aminoterminal region of Reelin, which is recognized by the function-blocking CR50 monoclonal antibody, is required for homodimerization, and clustered (but not monomeric) receptor-associated protein (RAP), an extracellular lipoprotein receptor ligand, or antibodies directed against the extracellular domains of Apoer2 or Vldlr were able to induce Dab1 tyrosine phosphorylation (Strasser et al., 2004). This was complemented by demonstrating that chemically inducible homodimerization of a Dab 1-FKBP12 fusion protein in heterologous cells was sufficient to induce receptor-independent tyrosine phosphorylation in a SFK-dependent manner (Strasser et al., 2004). Altogether, these studies support the hypothesis that Reelin can induce Apoer2/Vldlr-dependent signaling by clustering Dab1 on the cytoplasmic leaflet of the plasma membrane, bringing it into close proximity with SFK, without the need for an additional coreceptor.

### Signaling Events Downstream of Dab1

Here, we will focus on molecular events that regulate Dab1 activation and turnover (and thereby the duration of the Reelin signal) by modulating Src kinase activity, and the Reelin-dependent formation of phospho-Dab1-mediated scaffolding complexes that activate downstream signaling cascades. The Dab1-dependent inside-out activation of other transmembrane receptors involving Rap1 will be discussed in the next chapter.

#### Feedback Regulation of Reelin Signaling by Dab1 Degradation

Previous studies demonstrated that tyrosine-phosphorylated Dab1 potentiates SFK activation by a positive feedback mechanism (Bock and Herz, 2003), which is limited by the Cullin 5 and SOCS/Rbx2-dependent ubiquitinylation and proteasomal degradation of tyrosine-phosphorylated, activated Dab1 (Arnaud et al., 2003a; Bock et al., 2004; Feng et al., 2007; Simó et al., 2010; Simó and Cooper, 2013; Lawrenson et al., 2015). This places the SFK both upstream and downstream of Dab1. Of note, expression of a mutant Dab1 protein that was phosphorylatable but partly resistant to Cullin-induced degradation in electroporated embryonic brains caused overmigration of cortical neurons, indicating that Dab1 degradation is an essential part of the Reelin-dependent regulation of neuronal positioning in the developing cortical plate (Simó et al., 2010). Accordingly, positioning defects in mice with a conditional deletion of Rbx2 in neuronal progenitors were partially rescued by reducing Dab1 levels through Dab1 heterozygosity, which suggests that accumulation of (phosphorylated) Dab1 by the inability to turn the Reelin signal off causes migration defects (Simó and Cooper, 2013). Conditional deletion of the GTPase-activating protein (GAP) tuberin (Tsc2), which is mutated in the genetic disorder tuberous sclerosis, leads to neuronal migration defects in the cortex, hippocampus and cerebellum that are reminiscent of but not identical with the *reeler* phenotype (Moon et al., 2015). The defects were traced to a defect in Reelin-Dab1-dependent signaling, which is caused by increased activation of mTor kinase and subsequent aberrant expression of the ubiquitin ligase Cullin 5, decreased levels of activated Fyn and phosphorylated Dab1, and increased total Dab1 levels, a biochemical hallmark of defective Reelin-Dab1 signaling (Rice et al., 1998). Interestingly, Reelin also activates mTor in an Akt-dependent manner to regulate growth and branching of hippocampal neurons (Jossin and Goffinet, 2007). An additional link between Reelin and mTor signaling is suggested by the reciprocal regulation of Golgi morphology by Reelin-Dab1 and Lkb1 signaling (Matsuki et al., 2010), which also inhibits mTor by activating Tsc2.

#### The Relevance of Different Tyrosine Phosphorylation Sites of Dab1

Molecular targets downstream of Dab1 depend on the phosphorylated tyrosine residues. By generating mice with tyrosine to phenylalanine exchanges either at amino acids 185 and 198/200 (ab) or at positions 220 and 232 (cd) of Dab1 (Feng and Cooper, 2009) provided *in vivo* evidence for a dual role of Dab1 both as a SFK activator and a phosphorylation-dependent scaffold for the assembly of downstream signaling complexes. The *Dab1*^ab^ and *Dab1*^cd^ homozygous mice displayed abnormal development of the neocortex and hippocampus, with intermediate cerebellar phenotypes when compared with mice lacking all five tyrosine phosphorylation sites (Howell et al., 2000). Mice hemizygous for each of the mutant alleles (*Dab1*^ab/cd^) showed no morphological brain abnormalities, which indicates that the two sites have individual functions that are required together to support normal brain development. Specifically, the ab site is required for SFK activation (Arnaud et al., 2003b; Bock and Herz, 2003), Dab1 phosphorylation and degradation, and PI3 kinase activation (Beffert et al., 2002; Bock et al., 2003), whereas phosphorylation of the cd site is important for the assembly of Crk/CrkL signaling complexes as well as for the interaction with other adapter proteins such as Nck2 or Crk family members (Pramatarova et al., 2003; Chen et al., 2004; Huang et al., 2004). Recently, Lawrenson et al. (2015) described a role for the tyrosyl residue at position 300 of Dab1, in concert with Y200, for the interaction with SOCS6 and 7, two SH2-containg proteins that are components of the ubiqutin-E3 ligase complex which is responsible for the proteasomal degradation of phosphorylated Dab1. Mice lacking SOCS6 and SOCS7 display a cortical layer inversion phenotype reminiscent of *reeler* mice and express increased levels of phosphorylated Dab1; however, Reelin-dependent phosphorylation at the Y300 site has not been directly demonstrated yet.

A requirement for PI3 kinase and Akt for normal cortical plate development was demonstrated in an organotypic brain slice culture assay by using chemical inhibitors (Bock et al., 2003; Jossin and Goffinet, 2007), although dominant-negative (DN) kinase-inactive Akt1 expressed under control of a neuron-specific *Dcx* promoter and electroporated into embryonic mouse brain at E12.5 did not affect glia-independent somal translocation (Franco et al., 2011). As would be expected, further branching of the signaling cascade leads to the activation of effectors like cofilin (Chai et al., 2009, 2016), which will not be discussed in detail here. Interestingly, the somatic activation of another Akt isoform, Akt3, in focal malformations of cortical development is responsible for non-cell autonomous cortical migration defects, which lead to drug-resistant epilepsy (Baek et al., 2015). Here, the migration defect is caused by Foxg1-mediated ectopic misexpression of Reelin.

The scaffolding function of Dab1 phosphorylated at the cd sites is essential for normal development. This is underscored by the *reeler*-like phenotype of mice where the adapter proteins Crk and CrkL were simultaneously deleted in the developing nervous system (Park and Curran, 2008). Since they are located downstream of phosphorylated Dab1 in the Reelin signaling cascade, Dab1 protein levels were not increased in the brains of Crk/CrkL double-mutant mice. Reelin-induced phosphorylation not only of the Crk/CrkL-binding guanine nucleotide exchange factor (GEF) C3G, which activates the small GTPase Rap1 (Gotoh et al., 1995), but also of the serine/threonine kinase Akt/protein kinase B (PKB) at position 473 was inhibited in Crk/CrkL-deficient neurons (Park and Curran, 2008). The latter might be a consequence of a loss of the direct interaction of the PI3K regulatory subunit p85 with Crk, which was shown in T lymphocytes (Gelkop et al., 2001).

#### Regulation by Constrained Localization and Differential Splicing of Dab1

In cultured neurons, Dab1 and Apoer2 are enriched in the distal dendrite of neurons (Howell et al., 1999b; Leemhuis et al., 2010), where Reelin stimulation leads to a localized increase of overall tyrosine phosphorylation (Beffert et al., 2002). The localized action of Reelin signaling is important for the activity-dependent enrichment of HCN1 and GIRK1 ion channels in the distal dendritic tuft of hippocampal CA1 and neocortical layer V pyramidal neurons, thereby modulating the molecular specification of the distal dendritic compartment (Kupferman et al., 2014).

Another level of regulation may be provided by differential splicing of the *Dab1* gene (Bar et al., 2003; Gao and Godbout, 2013). In mice lacking the RNA-binding protein Nova2 a migration defect of late-born cortical neurons was observed. This phenotype was dependent on the overrepresentation of an alternatively spliced form of Dab1 during a critical time window between E14.5 and E16.5, which is normally suppressed by the splicing factor Nova2 (Yano et al., 2010).

### Dab1-Dependent Inside-Out Activation of Cell Surface Receptors by Reelin

Several studies addressed the role of Rap1, which is activated through the Reelin-Dab1-Crk-C3G pathway (Ballif et al., 2004), during neocortical development. C3G hypomorphic mice display cortical migration defects resulting in a failure of preplate splitting similar to *reeler* mice, and defects in radial glial processes (Voss et al., 2008). Inactivation of neuronal Rap1 by *in utero* electroporation of the gene encoding *Rap1Gap*, a GAP that specifically inactivates Rap1 by stimulating its GTP hydrolysis, or *Rap1a* shRNA, disrupted glia-independent somal translocation of migrating neurons. This effect was dependent on neuronal Cadherin-2 (NCAD, Cdh2), a transmembrane protein that mediates cell-cell adhesion. Cdh2 overexpression rescued the migration defect caused by Rap1GAP overexpression, placing it downstream of Rap1 as an example of inside-out activation of a cell surface receptor. However, the *Dab1* null phenotype was not rescued by Cdh2 overexpression, which underscores the pleiotropic nature of Reelin-Dab1 signaling affecting different developmental stages, cellular and molecular levels of action (Franco et al., 2011). This is exemplified by two other important studies using *in utero* electroporation of embryonic brains, which confirmed the importance of Reelin-dependent Rap1 activation for neuronal migration in the developing neocortex (Jossin and Cooper, 2011; Sekine et al., 2012). Sekine et al. (2012) found that Cdh2 overexpression did not fully rescue the Rap1 suppression phenotype and provided evidence for an additional role of Dab1-Crk-C3G-Rap1 dependent inside-out activation of integrin β1-α5 receptors for the terminal translocation step of migrating neurons. Moreover, they demonstrated a role for Akt in the regulation of the terminal translocation step, which was rescued by simultaneous coexpression of constitutively active integrin α5 and Akt, but not by either vector alone. It should be mentioned, though, that neuron-specific conditional inactivation of β1 integrin using NEX-Cre mice did not affect the formation of cortical cell layers (Belvindrah et al., 2007). A cell-autonomous role of Dab1 phosphorylation at tyrosine residues 220 and 232 for the detachment of neurons from radial glia was shown, which depends on the downregulation of neuronal integrin α3 levels (Sanada et al., 2004). This provides an example for Reelin-dependent inside-out modulation of a cell surface receptor (Figure 2B) by downregulation of its activity.

Jossin and Cooper (2011) showed that Rap1 regulated the membrane localization of N-cadherin, which was required for the transition from multipolar to bipolar migration in the lower intermediate zone. Of note, migration defects of neurons overexpressing a signaling-incompetent, dominant-negative (DN) Vldlr construct were only partially rescued by transfection with a constitutively active form of Rap1, whereas coexpression of Akt and activated Rap1 overcame the effect of DN-Vldlr (Jossin and Cooper, 2011). In the marginal zone, Reelin-dependent Rap1 signaling influences the interaction of translocating neurons with Reelin-secreting Cajal-Retzius cells by facilitating Cdh2 recruitment to nectin-based adhesion sites, with the Rap1 effector Afadin and its binding partner p120-Catenin serving as a molecular link between the activation of Rap1 and Cdh2 (Gil-Sanz et al., 2013). However, Cdh2 is not only required for the multipolar-bipolar transition and terminal translocation step of cortical neuronal migration but also involved in mediating heterophilic interactions of migrating neurons with radial glia during the locomotion mode of migration, which depends on the regulation of vesicle trafficking by Rab GTPase proteins (Kawauchi et al., 2010). The importance of Rap1 is underlined by the phenotype of conditional knockout mice, which show a complete loss of cortical lamination as a consequence of the loss of radial glial and neuronal polarity (Shah et al., 2016). Knockdown of the RasGAP Dab2IP, a Dab1-interacting protein (Homayouni et al., 2003), has been reported to influence the positioning of later-born cortical neurons by activating Rap1 and integrin signaling (Qiao and Homayouni, 2015), and to regulate the multipolar-bipolar transition in the intermediate zone (Lee et al., 2012). However, no complete *reeler*-like phenotype was reported for conventional Dab2IP (AIP1) knockout mice (Zhang et al., 2008). Other small GTPases like Cdc42 and possibly Rac1 are involved in more specific functions of Reelin during the differentiation of postmitotic neurons (Leemhuis et al., 2010; Jossin, 2011; Leemhuis and Bock, 2011; Meseke et al., 2013; Pasten et al., 2015), where fine-tuning is achieved by the involvement of various GEFs (Rossman et al., 2005).

### Role of Dab1 in Receptor Trafficking

Dab1 is a protein with several features of an endocytic accessory factor (Merrifield and Kaksonen, 2014) and might therefore be involved in the regulation of receptor trafficking. This cellular function of Dab1 is relevant to understanding important aspects of Reelin signaling via both canonical and non-canonical Reelin receptors. The aminoterminal phosphotyrosine binding/PTB domain preferentially interacts with the non-phosphorylated NPXY tetra-amino-acid motif (Herz et al., 1988; Chen et al., 1990) in the intracellular tails of transmembrane receptors, including Apoer2 and Vldlr but also the amyloid precursor protein family members and integrins (Rice et al., 1998; Trommsdorff et al., 1998; Howell et al., 1999b; reviewed in Stolt and Bock, 2006; Yap and Winckler, 2015), and also mediates membrane localization by interacting with phosphoinositides (Stolt et al., 2003, 2005; Huang et al., 2005; Xu et al., 2005). In addition, it contains a clathrin binding site within the carboxyterminal domain, which is involved in the regulation of cortical development, since mice expressing only one copy of a carboxyterminally truncated, hypomorphic p45 isoform of Dab1 display migration defects of late-born cortical neurons (Herrick and Cooper, 2002). Morimura et al. (2005) reported data from experiments in cortical neurons and heterologous cells suggesting that tyrosine-phosphorylated Dab1 is recruited to the plasma membrane after Reelin stimulation and that phosphorylation of Dab1 initiates intracellular trafficking of Reelin.

Interaction with Dab1 increases cell surface levels and proteolytic processing of Apoer2 and APP independent of its tyrosine phosphorylation and increases cleavage of extracellular receptor domains (Hoe et al., 2006b). Interaction of tyrosine-phosphorylated Dab1 with the endocytic adapter protein CIN85 in Reelin-treated neurons might contribute to the sorting of Dab1-receptor complexes to early endosomes (Fuchigami et al., 2013). Another regulator of endosomal receptor cycling, sorting nexin 17 (Snx17), binds to the intracellular domain of lipoprotein receptors including Apoer2 (Stockinger et al., 2002), and was reported to contribute to Reelin-induced Apoer2 trafficking, processing and Dab1 dependent signaling (Sotelo et al., 2014). It should be mentioned that based on studies in heterologous cells it was also suggested that Dab1 has two nuclear localization signals (NLS) and two nuclear export signals (Honda and Nakajima, 2006, 2016) and could therefore act as a nucleocytoplasmic shuttling protein. Tyrosine phosphorylation did not affect the intracellular distribution of Dab1 (Honda and Nakajima, 2006), arguing against a direct role of Dab1 in mediating Reelin-dependent regulation of transcription (Telese et al., 2015). *In utero* electroporation of Dab1 harboring a mutant NLS confirmed that excess cytoplasmic Dab1 inhibits neuronal migration (Honda and Nakajima, 2016).

### Crosstalk with p35/Cdk5 Signaling

Together, the above-mentioned studies provide important clues about the molecular mechanisms of Reelin’s contributions to different steps of neuronal layer formation in the developing neocortex. However, issues such as the the specific contribution of Reelin effectors in different responsive cell types, and the interaction with other signaling pathways (Figure 2F) are still insufficiently understood. Activation of Rap1 constitutes an example of signaling crosstalk with Cyclin-dependent kinase 5 (Cdk5) that is fairly well examined. Cdk5 is a serine/threonine kinase that is highly expressed in postmitotic neurons. Its essential role during brain development is obvious from the phenotype of *Cdk5* knockout mice, which display severe defects in laminated brain structures. Like Reelin, Cdk5 also modulates many aspects of neuronal maturation and synaptic transmission in the adult brain (reviewed by Dhavan and Tsai, 2001; Kawauchi, 2014; Shah and Lahiri, 2014). However, in contrast to *reeler* mice, the preplate splits normally during neocortical development (Gilmore et al., 1998), and the failure of Reelin to induce Cdk5 activation (Gilmore et al., 1998) as well as a series of genetic studies support the concept that Reelin and Cdk5 act in parallel rather than in a linear fashion to regulate layer formation in the developing brain (Ohshima et al., 2002; Beffert et al., 2004; Ohshima, 2015). Rap1 has been shown to be an important convergence point of both pathways. Whereas RapGEF1 (C3G) is activated by Reelin, RapGEF2 is phosphorylated and thereby activated by Cdk5 at Ser1124, which seems to be important for the transition from multipolar to bipolar morphology in the intermediate zone (Ye et al., 2014). Other possible intersection points of Reelin and Cdk5 signaling include collapsin response mediator proteins (CRMP), whose phosphorylation by the Reelin target Gsk3beta (Beffert et al., 2002) needs to be primed by prior Cdk5 phosphorylation (Uchida et al., 2005; Cole et al., 2006); the microtubule-associated phosphoprotein Tau (Sengupta et al., 1997; Li et al., 2006; Plattner et al., 2006), which is involved in the pathogenesis of neurodegenerative disease, and Dab1 itself (Keshvara et al., 2002; Ohshima et al., 2007), although evidence for the *in vivo* relevance of these interaction nodes is sparse. Expression of a carboxyterminally truncated form of Dab1 that lacks the Cdk5 phosphorylation sites rescues the Dab1 knockout phenotype, but mice hemizygous for the truncated gene (*Dab1*^p45/−^) display a unique migration defect in the neocortex, with normal preplate splitting as in the *Cdk5* knockout mice, and hippocampus (Herrick and Cooper, 2002).

### Non-Canonical Reelin Signaling Involving Apoer2/Vldlr, or Dab1

Noncanonical signaling can refer to the involvement of components other than Apoer2/Vldlr as cell surface receptors or Dab1 as intracellular signal transducer of Reelin, which are the essential components of the “classical” Reelin cascade, as outlined above. Indeed, several studies have been published that suggest a role for Reelin binding to its canonical receptors Apoer2 and/or Vldlr without involving Dab1 as the central intracellular mediator of this interaction. Other studies point to a requirement of Dab1 for the transmission of the Reelin signal without the involvement of Apoer2 or Vldlr (Figures 2D,E). Rossel et al. (2005) reported that Reelin is required for the second, radial glia-dependent migration step of hindbrain efferent neurons. This phenotype was also observed in Dab1-deficient scrambler mice but not in *Apoer2/Vldlr*-deficient mice, pointing to the involvement of another receptor for Reelin. Possible candidates would be transmembrane proteins that interact with Dab1 via their intracellular tails, e.g., integrins or amyloid precursor protein.

Conversely, a Reelin-dependent effect on the migration of early-generated interneurons in the olfactory bulb was described, which was defective in lipoprotein receptor-deficient mice but was not phenocopied in mice lacking Dab1 (Hellwig et al., 2012), suggesting that context-dependent alternative signal transduction mechanisms for Reelin exist. Still another scenario was described for hypothalamic gonadotropin releasing hormone (GnRH)-positive neurons, with a reduction in number and aberrant position of GnRH neurons in the hypothalamus of *reeler*, but not Dab1 or Apoer2/Vldlr-deficient neurons (Cariboni et al., 2005). A similar situation is found during lymphatic vascular development, which is defective in Reelin-deficient *reeler* mice but not in mice lacking Dab1 or both Apoer2 and Vldlr (Lutter et al., 2012). In none of these cases, the molecular mechanisms underlying the described Reelin-dependent phenotypes have been elucidated.

### Reelin-Independent Activation of Lipoprotein Receptor-Dab1 Dependent Signaling

Another “non-canonical” variation of classical Reelin signaling is the activation of the core signaling pathway by ligands other than Reelin itself. Reelin has been described to be important for the so-called chain migration of neuroblasts from the subventricular zone into the olfactory bulb along the rostral migratory stream (RMS; Hack et al., 2002). Here, Reelin induces the detachment of chain-migrating neurons, leading to a switch from chain migration to radial migration in the olfactory bulb. Apoer2 and Dab1 are expressed in the RMS, and Apoer2 and Vldlr seem to be involved in mediating the effect of Reelin on the detachment process (Hellwig et al., 2012). Because Reelin is not present in the RMS (Hack et al., 2002; Andrade et al., 2007), it was suggested that the involvement of Apoer2/Vldlr and Dab1 for proper neuroblast chain formation indicates the requirement for another lipoprotein receptor ligand (Andrade et al., 2007). Since the extracellular matrix (ECM) protein thrombospondin-1 (Tsp1) is expressed in the RMS and Tsp1-deficient mice have a wider and less compact RMS architecture it was examined whether Tsp1 might act as a ligand for Apoer2 and Vldlr, which was shown to be the case (Blake et al., 2008). Tsp1 binding to the receptors was competitive to both Reelin and receptor-associated protein (RAP), a chaperone and universal ligand for LDL receptor family members (Herz et al., 1991), and treatment with Tsp1 induced the tyrosine phosphorylation of Dab1 in primary neurons, probably by promoting receptor multimerization (Blake et al., 2008). Surprisingly, however, other key features of canonical Reelin signaling, such as ligand-induced Dab1 degradation were not observed, and in an *in vitro*-based matrigel assay Tsp1 stabilized neuronal precursor chains (Blake et al., 2008), instead of dissolving them like Reelin does (Hack et al., 2002). The molecular basis of these differences in downstream signaling via Apoer2 and Dab1 remains to be identified and might involve differences in the phosphorylation of the various Dab1 tyrosyl residues, or the different activation of modulating signaling pathways by coreceptors. Another ECM protein that has been reported to bind to Apoer2 was F-spondin (Hoe et al., 2005). The interaction site was mapped to the aminoterminal thrombospondin domains of F-spondin, which are located in the carboxyterminal half of the protein, and was inhibited by the LDL receptor family chaperone RAP, whereas the aminoterminal Reelin and spondin domains bound to the amyloid precursor protein (APP). These interactions were reported to increase the cell surface expression of both receptors and modulated their proteolytic processing (Ho and Sudhof, 2004; Hoe et al., 2005). In chicken ciliary ganglion (CG) neurons, F-spondin induced tyrosine phosphorylation of Dab1 (Peterziel et al., 2011). Surprisingly, this was likely mediated through binding of F-spondin to APP (Figure 2D), since Apoer2 and Vldlr were barely expressed in the GC cells, and the lipoprotein receptor antagonist RAP did not block the neurotrophic effect of F-spondin on GC cells, which was shown to depend on Dab1 phosphorylation (Peterziel et al., 2011).

In another study that was based on the observation of an olfactory bulb layering defect in insulin-like growth factor 1 (IGF1)-deficient mice an effect of IGF1 on Dab1 phosphorylation in OB cell cultures was reported (Hurtado-Chong et al., 2009). However, the phosphosite-specific Dab1 antibodies used in this study were confirmed for overexpresssing cells only and are not specific in primary cortical cultures, and the results should therefore be validated by Dab1 immunoprecipitation followed by phosphotyrosine immunoblotting, or by using OB cells from Dab1-deficient animals. Other ligands that have been described to induce tyrosine phosphorylation of Dab1 via lipoprotein receptor-dependent signaling include fibrillar prion protein fragment, which also reduces total Dab1 levels after prolonged treatment (Gavín et al., 2008), and clusterin/apolipoprotein J, which is present in the adult subventricular zone and might be involved in neurogenesis and neuroblast chain formation (Leeb et al., 2014). Another effect mediated by Apoer2 which does not depend on Reelin was described in the monocytic cell line U937: Activated protein C (APC) induced Dab1 tyrosine phosphorylation and Akt-dependent Gsk3beta phosphorylation in a RAP-sensitive manner (Yang et al., 2009; Sinha et al., 2016), which contributed to APC’s anticoagulant activity after endotoxin stimulation *in vitro*. Vldlr did not bind to APC as determined by surface plasmon resonance or solid-phase binding assays (Yang et al., 2009). It remains to be determined if Reelin, which is present in large amounts in the plasma (Smalheiser et al., 2000), has similar effects on circulating blood cells. Vascular endothelial growth factor (VEGF)-induced tyrosine phosphorylation of Dab1 mediated by the receptor tyrosine kinase Flk1 (VEGF receptor-2) in cortical neurons has been reported using a phospho-specific DAB1 antibody (Howell et al., 2013). Again, this finding should be confirmed by Dab1 immunoprecipitation followed by immunoblotting with a phosphotyrosine antibody.

It should be mentioned that selenoprotein P (Sepp1) has been described as an Apoer2 ligand that does not interfere with canonical lipoprotein receptor-Dab1 signaling. Instead, its endocytosis via Apoer2 is essential for selenium supply in the brain and testis (Olson et al., 2007; Masiulis et al., 2009), and is mediated by the Apoer2 beta-propeller domain instead of the ligand binding domain (Kurokawa et al., 2014). Another Apoer2 and Vldlr ligand, the proprotein convertase Pcsk9, binds to the epidermal growth factor-like repeat A of LDL receptor family members next to the ligand binding domain and mediates the intracellular degradation of both receptors by rerouting them to a lysosomal pathway (Cohen and Hobbs, 2013). Whereas this effect was enhanced by the presence of Dab1 in heterologous cells (Poirier et al., 2008), its physiological relevance for Reelin signaling in the brain is unclear (Liu et al., 2010; Kysenius et al., 2012).

### Selective Functions of the Canonical Reelin Receptors Apoer2 and Vldlr

Apoer2 and Vldlr are close relatives of the LDL receptor and share a high degree of homology, although important structural differences exist, which are reviewed elsewhere (Bock and Herz, 2008; Reddy et al., 2011). Both receptors bind Reelin as high-affinity receptors, with slightly different binding affinities (Andersen et al., 2003; Benhayon et al., 2003), and can mediate tyrosine phosphorylation of Dab1 as well as activation of Dab1-dependent downstream targets like Akt without the requirement of the presence of the other receptor, as was first shown by using cultured cortical neurons from either *Apoer2* or *Vldlr* single-knockout mice (Beffert et al., 2002). However, the single-knockout mice display strikingly different phenotypes (e.g., Trommsdorff et al., 1999; Weeber et al., 2002; Hack et al., 2007), which is largely explained by the non-overlapping spatiotemporal and subcellular expression pattern of both receptors (Perez-Garcia et al., 2004; Hirota et al., 2015). In addition, differences exist with regard to the subcellular distribution, especially the recruitment to cholesterol-rich microdomains of the plasma membrane (Sun and Soutar, 2003; Mayer et al., 2006), which have important functions in regulating signal transduction and receptor trafficking (reviewed in Lingwood and Simons, 2010). Also, differences in their capacity to mediate endocytosis and degradation of bound ligands likely contribute to their different biological functions (Li et al., 2001; Beffert et al., 2006a; Chen et al., 2010; Duit et al., 2010). Another possible mechanism would be the receptor-specific interaction with different adapter proteins. Important aspects of Apoer2’s function as Reelin receptor depend on its differential splicing. The O-linked sugar domain of Apoer2 in close proximity of the transmembrane domain is encoded by a separate exon and required for the extracellular cleavage of Apoer2, which precedes its gamma-secretase mediated proteolytic processing (May et al., 2003b). Mice lacking this exon showed increased Apoer2 abundance in the brain, which was associated with altered synaptic receptor function (Wasser et al., 2014). Activity-dependent alternative splicing of the intracellular exon 19 of Apoer2, which encodes a proline-rich insert that mediates biochemical and functional interaction with NMDA receptors at the synapse (Beffert et al., 2005; Hoe et al., 2006a), represents an additional important means to fine-tune biological responses to Reelin signaling in a context-dependent manner. Scaffold proteins of the JIP and MINT families also bind to Apoer2, but not to Vldlr (Figure 2E), in an exon 19-dependent manner (Gotthardt et al., 2000; Stockinger et al., 2000; Verhey et al., 2001; He et al., 2007; Minami et al., 2010). The JIP-JNK recruiting function of the Apoer2 isoform including the proline-rich insert is required for protection against loss of corticospinal neurons (CSN) during normal aging, whereas the same splice form promotes lesion-induced cell death of CSN, as was elegantly shown *in vivo* by using knockin mice with selective alterations of the Apoer2 intracellular domain (Beffert et al., 2006b). The Dab1 binding site was not involved, suggesting Reelin signaling-independent functions of the Apoer2 intracellular domain. Direct phosphorylation of JNK was demonstrated in hippocampal neurons treated for 1 h with recombinant Reelin. This was blocked by either PI3K inhibition or pertussis toxin, suggesting involvement of heterotrimeric G protein and crosstalk with G-protein coupled receptor signaling (Cho et al., 2015).

On the other hand, Vldlr-specific Reelin signaling responses (Figure 2E) have been described. Following demonstration that Reelin and LIS1, the gene product of the *Pafah1b1* gene, genetically and biochemically interact in a phospho-Dab1-dependent manner (Assadi et al., 2003) it was shown that the α1 and α2 catalytic subunits of the Pafahb complex selectively bind the NPXYL motif in the intracellular Vldlr domain (Zhang et al., 2007) and differentially interact with either tyrosine phosphorylated or non-phosphorylated Dab1, which modulates the effect of Reelin-Dab1-Lis1 signaling on microtubule dynamics (Assadi et al., 2008; Zhang et al., 2009). In a recent study, it was reported that Vldlr-dependent Ras signaling affects dendritic spine formation in hippocampal neurons (DiBattista et al., 2015). The authors used a recombinant Reelin fragment, which decreased the biochemical interaction of Vldlr with the GEF RasGRF1; however, it remains to be determined if full-length Reelin exerts the same effect.

The same murine Reelin fragment encompassing amino acids 1221–2661 (i.e., corresponding to the central fragment consisting of the Reelin repeats 3–6 that is able to bind to Apoer2 and Vldlr and to induce Dab1 phosphorylation in cultured neurons (Jossin et al., 2004)) was used to demonstrate coclustering of Apoer2 with other receptors (Divekar et al., 2014). This fragment includes a critical cysteine residue at position 2101 that is required for covalent multimerization and efficient Dab1 phosphorylation (Yasui et al., 2011). Coprecipitation of Apoer2 and Vldlr after stimulation with the Reelin fragment was not observed in heterologous cells and primary neurons, leaving open the question if Reelin induces its two main receptors to form heteroclusters to a significant extent.

### Activation of Pathways that Modulate Transcription

Another pivotal protein kinase cascade that relays extracellular signals from the cell surface into cells is the Erk (extracellular signal-regulated kinases) pathway, which was reported to be activated by Reelin in a Src- and Dab1-dependent manner (Simó et al., 2007). Whereas other studies could not detect Erk phosphorylation after stimulation with Reelin-conditioned medium (Ballif et al., 2003; Cho et al., 2015), the activation of Erk in primary neurons was confirmed using highly purified Reelin (Lee et al., 2014; Telese et al., 2015). In contrast to the aforementioned study (Simó et al., 2007), this activation did not depend on Dab1 and was not inhibited by recombinant RAP, a chaperone that blocks binding of extracellular ligands to LDL receptor family members, which suggests involvement of a different Reelin receptor. This is supported by the observation that the lipoprotein receptor-binding central fragment of Reelin was not sufficient to induce Erk phosphorylation (Lee et al., 2014). Reelin-induced Erk activation was accompanied by increased transcription of immediate early genes including *Egr1* and *Arc* (Simó et al., 2007; Lee et al., 2014). In addition, it was demonstrated that Reelin directly potentiates glutamate-induced NMDA receptor-dependent calcium influx (Chen et al., 2005). This was mediated via activation of Src kinases, Dab1 and NMDAR phosphorylation and induced the phosphorylation of cAMP-responsive element binding protein (Creb) at serine 133 (Chen et al., 2005), a transcription factor that modulates many aspects of neuronal development, plasticity and behavior in response to PI3K/Akt and Erk activation (Lonze and Ginty, 2002). Together, these findings suggested that regulation of gene transcription contributes to Reelin’s multiple effects on brain development and function. Indeed, transcriptomic profiling of mature cortical neurons confirmed that Reelin induces the expression of synaptic activity-regulated genes in a Src- and NMDAR-dependent manner (Telese et al., 2015). This involved Reelin-induced epigenomic changes that were sensitive to gamma-secretase inhibition, possibly involving nuclear translocation of the intracellular domain of Apoer2 (Telese et al., 2015), which is released by gamma-secretase activity in neurons (May et al., 2003b; Hoe and Rebeck, 2005; Wasser et al., 2014).

Another means of gamma-secretase dependent transcriptional regulation involving Reelin is its crosstalk with Notch signaling, a pathway that regulates many aspects of neural development, cell fate specification, neuronal survival and synaptic plasticity (reviewed by Ables et al., 2011; Pierfelice et al., 2011). A link between Notch and Disabled was first shown in *Drosophila* (Giniger, 1998). In mice, activation of Notch receptors leads to the gamma-secretase mediated release and nuclear localization of their intracellular domains (NICD), which regulates the transcription of target genes. In *reeler* mice, levels of the NICD and its target genes *Hes1* and *Hes5* were reduced, and overexpression of the NICD by electroporation rescued the neuronal migration defect in *reeler* mice or caused by overexpression of the Dab1-5F mutant, which cannot be tyrosine-phosphorylated at Y185, Y198/200, Y220 and Y232 (Hashimoto-Torii et al., 2008). This places NICD, which biochemically interacts with Dab1 (Hashimoto-Torii et al., 2008; Keilani and Sugaya, 2008), downstream of Dab1. Apart from its effect on neocortical migration, the interaction of Reelin and Notch affects the radial glial characteristics of progenitor cells (Keilani and Sugaya, 2008; Sibbe et al., 2009; Lakomá et al., 2011), including expression of brain lipid binding protein (Blbp), an effector of both pathways (Gaiano et al., 2000; Hartfuss et al., 2003). In a recent study it was shown that both Erk and Creb phosphorylation are reduced in the hippocampi of conditional Notch1-deficient mice, and suggested that Notch1 is also required for NMDAR-mediated Reelin signaling at the synapse (Brai et al., 2015).

### Non-Canonical Reelin Receptors

The inside-out activation of integrin receptors by Reelin-Dab1-Crk-Rap1 dependent signaling has been discussed above; here, integrin transmembrane proteins act as downstream effectors of Reelin signaling via the canonical lipoprotein receptor-Dab1-mediated pathway (Sekine et al., 2012). Direct binding of Reelin via its aminoterminal region to the extracellular domain of α3 β1 integrins has also been shown (Dulabon et al., 2000; Schmid et al., 2005). In addition, Dab1 interacts with the NPXY motif in the cytoplasmic tails of beta integrins (Calderwood et al., 2003; Schmid et al., 2005). In α3 β1 integrin-deficient cortical neurons Reelin-induced phosphorylation was not affected, but interestingly, Dab1 levels were downregulated in α3 integrin-deficient brains (Dulabon et al., 2000), as opposed to the characteristic upregulation of Dab1 in mice with genetic defects in the canonical lipoprotein receptor-dependent Reelin signaling cascade (see above). The preplate forms normally in the cortex of α3-deficient mice (Schmid et al., 2004), whereas inactivation of β1 integrin in radial glial cells, which are both neural and glial progenitor cells (reviewed by Dimou and Götz, 2014), develop a disorganized cortex with Cajal-Retzius cell heterotopia (Graus-Porta et al., 2001). Neuron-specific inactivation of β1 integrin produced no phenotype (Belvindrah et al., 2007), which suggests that Reelin-integrin binding alone is not responsible for the neuronal migration defect seen in *reeler* mice (Magdaleno and Curran, 2001) and that β1 integrin expression is pivotal for radial glial function (Forster et al., 2002; Radakovits et al., 2009).

Chronic treatment of hippocampal neurons with Reelin altered the subunit composition of synaptic NMDA receptors, which involved β1 integrin activity as shown by using function-blocking antibodies. The lipoprotein receptor antagonist RAP had no effect (Groc et al., 2007), ruling out inside-out activation of integrins via lipoprotein receptors as underlying signaling mechanism. Moreover, a presynaptic effect of Reelin on neurotransmitter release, which was blocked by a cyclic integrin-inhibiting arginylglycylaspartic acid (RGD) peptide (Ruoslahti, 1996), has been reported (Hellwig et al., 2011). In mouse brain synaptosome preparations Reelin enhanced the local translation of *Arc* mRNA (Dong et al., 2003), an immediate early gene that regulates synaptic plasticity (Shepherd and Bear, 2011), and this was inhibited by echistatin, an RGD-containing peptide that functions as a competitive integrin receptor antagonist (Gan et al., 1988). Hence, integrins might modulate the functions of Reelin at the synapse. However, a selective increase of spontaneous neurotransmission by Reelin was reported to depend both on Vldlr and Apoer2, which was also expressed presynaptically, and PI3 kinase activity, and required the vesicular SNARE protein Vamp7 (Bal et al., 2013). Apart from brain-specific roles, integrins are prime candidate receptors for Reelin functions in “peripheral” organs (Lin et al., 2016), many of which do not express detectable amounts of essential components of the canonical signaling cascade.

#### Reelin as a Ligand for Amyloid Precursor Protein (APP)

The demonstration that the interaction of Reelin with lipoprotein receptors at the adult synapse enhances long-term potentiation through Dab1- and Fyn-dependent phosphorylation of NMDA receptors (Weeber et al., 2002; Beffert et al., 2005; Chen et al., 2005) suggested that Reelin signaling in the adult brain modulates learning and memory performance, which has subsequently been demonstrated *in vivo* using different experimental approaches including conditional knockout of Reelin, Dab1, or Reelin overexpression in adult mice (Brosda et al., 2011; Rogers et al., 2011, 2013; Trotter et al., 2013; Pujadas et al., 2014; Lane-Donovan et al., 2015; Imai et al., 2016). As LDL receptor family members are main receptors for Apo E (reviewed by Herz and Willnow, 1994; Hussain et al., 1999), whose E4 variant is a strong genetic risk factor for the sporadic form of Alzheimer disease (AD; (Strittmatter et al., 1993), a causal connection between disturbed Reelin signaling and neurodegeneration has been proposed ever since Apoer2 and Vldlr were first described as Reelin receptors (Cooper and Howell, 1999; Bothwell and Giniger, 2000; Herz, 2001). This view, summarized in several reviews (e.g., Rogers and Weeber, 2008; Krstic et al., 2013; Lane-Donovan et al., 2014) is supported by a multitude of *in vitro* and *in vivo* studies, which collectively suggest that Reelin has an overall neuroprotective role in the adult brain. The amyloid-beta peptide, which is considered to be central for the development and progression of AD (Selkoe, 2000), is generated by sequential proteolytic cleavage from a type I transmembrane receptor named APP (O’Brien and Wong, 2011). Importantly, the inhibition of hippocampal long-term potentiation (LTP) by soluble amyloid-beta oligomers (reviewed by Selkoe, 2008) is overcome by coapplication of Reelin in acute slices (Durakoglugil et al., 2009), and selective retardation of cell surface recycling of endocytosed Reelin-ApoE receptor complexes by ApoE4-containing lipoproteins at least partially explains the negative effect of this isoform on synaptic function (Chen et al., 2010). Inactivation of Reelin in the adult brain by tamoxifen-inducible conditional gene knockout precipitated amyloid-beta neurotoxicity in transgenic mice overexpressing an AD-associated mutant form of APP (Lane-Donovan et al., 2015). In a reverse approach, inducible overexpression of Reelin was shown to overcome toxic effects of amyloid-beta (Pujadas et al., 2014). In this study, Reelin delayed the formation of amyloid fibrils, the main constituents of senile plaques, which were previously shown to accumulate Reelin both in animal models of AD and during normal aging (Wirths et al., 2001; Knuesel et al., 2009). Importantly, however, loss of Reelin in the conditional knockout model did not accelerate amyloid plaque deposition at the studied age of 7 months (Lane-Donovan et al., 2015). This rules out the possibility that Reelin’s protective role at the synapse is predominantly mediated by regulating plaque abundance.

Beside its function as a preproprotein whose processing generates biologically active soluble fragments (Haass et al., 2012) and a transcriptionally active intracellular domain (Müller et al., 2008) the role of the full-length amyloid precursor protein as a cell surface receptor is being increasingly recognized (Deyts et al., 2016), although its normal physiological functions remain largely unknown. Reelin has been reported to be one of several candidate ligands of full-length APP (Hoe et al., 2009) and modulates the proteolytic processing of APP *in vivo* and *in vitro* (Kocherhans et al., 2010; Rice et al., 2013), partly through the interaction of APP with Dab1 (Rice et al., 1998; Trommsdorff et al., 1998; Howell et al., 1999b), Fe65, and lipoprotein receptors (Hoe et al., 2006b, 2008; Parisiadou and Efthimiopoulos, 2007; Kwon et al., 2010; Minami et al., 2011). The direct binding of Reelin to the extracellular domain of APP involves its central fragment, which also interacts with the ligand binding domains of Apoer2 and Vldlr (Hoe et al., 2009). A possible function of this interaction, which was reported to involve α3 β1 integrins, might be the regulation of neurite outhgrowth (Hoe et al., 2009). An excess of the APP intracellular domain blocked the inhibitory effect of cell surface-immobilized Reelin on neurite outgrowth, possibly by sequestering Dab1 in the nucleus (Hoareau et al., 2008). The genetic interaction of *Dab1* and *App*, and the effect of *App* knockdown or overexpression on neuronal migration (Young-Pearse et al., 2007; Pramatarova et al., 2008) support additional functions for Reelin-APP and APP-Dab1 interactions during neurodevelopment.

#### Interaction with Eph Receptors

Mice lacking members of the EphB transmembrane tyrosine kinase receptor family, which mediate a variety of interactions regulating brain development and function (reviewed by Klein, 2004; North et al., 2013), display deficits in hippocampal morphogenesis (Catchpole and Henkemeyer, 2011; Bouché et al., 2013). This observation led to the identification of EphB proteins as Reelin receptors. Binding to the extracellular domain of EphB2 is mediated by the aminoterminal part of Reelin and induces EphB forward signaling (Figure 2D) in heterologous cells expressing EphB2 and in primary neurons (Bouché et al., 2013). Principally, the interaction with the aminoterminal domain of Reelin allows for the simultaneous binding of lipoprotein receptors or APP, which interact with Reelin via its central fragment. The composition of the putative supramolecular Reelin-receptor complex might be further modulated through regulated proteolytic cleavage of Reelin (Lambert de Rouvroit et al., 1999; Jossin et al., 2007; Kohno et al., 2009; Krstic et al., 2012; Tinnes et al., 2013; Koie et al., 2014; Trotter et al., 2014; Sato et al., 2016) and gamma-secretase dependent intramembrane proteolysis of its receptors (Haass and De Strooper, 1999; May et al., 2003b; Hoe and Rebeck, 2005, 2008; Litterst et al., 2007; Xu et al., 2009; Bouché et al., 2013; Wasser et al., 2014), which opens up new avenues of crosstalk with other important neuronal signaling receptor systems (Larios et al., 2014).

Whereas the hippocampal defect in EphB1; EphB2 compound deficient mice that could be attributable to defective Reelin-mediated EphB forward signaling is limited to the CA3 region, other functions of this non-canonical Reelin-receptor interaction in the central nervous system might relate to the positioning of Cajal-Retzius cells by EphB-dependent contact repulsion during brain development (Villar-Cerviño et al., 2013), or the fine-tuning of NMDA receptor signaling at the synapse (Cissé et al., 2011; Nolt et al., 2011). The interaction might also be relevant to organs and tissues outside of the nervous system (Jung et al., 2011).

## Summary and Perspective

The discovery of the canonical linear lipoprotein receptor/Dab1-dependent Reelin signaling cascade has enabled us to decipher many of the cellular and molecular mechanisms underlying Reelin’s multiple functions in the developing and adult brain, and is now one of the best-characterized signaling pathways involved in shaping the developing brain (Ayala et al., 2007). However, even for well-established Reelin targets, many open questions regarding their exact functional relevance remain, which can be attributed to the pleiotropic actions of Reelin at different stages of development, different Reelin-responsive cells, different requirements for specific Reelin domains depending on the developmental stage (Kohno et al., 2015), functional redundancies of signaling components, posttranslational modifications of Reelin (Botella-López et al., 2006), and various technical obstacles (Baek et al., 2014). The biological functions of non-canonical Reelin signaling cascades, as outlined in detail above, are even less well defined and require further investigation.

Many of the non-canonical Reelin-receptor interactions are possibly related to the expression of Reelin outside the brain, which has first been acknowledged shortly after the Reelin gene was discovered (Ikeda and Terashima, 1997). Various tissues and organs contain Reelin at relatively high concentrations, including plasma, blood cells, liver and intestine (Smalheiser et al., 2000; Lugli et al., 2003; Underhill et al., 2003; García-Miranda et al., 2010; Böttner et al., 2014; Ding et al., 2016), and altered expression, glycosylation and processing of peripheral Reelin under pathophysiological conditions has been described (Botella-Lopez et al., 2008). Moreover, an association of (mostly reduced) Reelin expression and malignancy of various tumors has been reported, even in tissues that normally do not express Reelin, which suggests a possible role in the control of tumorigenesis and/or metastasis via unknown mechanisms (Wang et al., 2002; Sato et al., 2006; Perrone et al., 2007; Dohi et al., 2010; Stein et al., 2010; Okamura et al., 2011; Castellano et al., 2016; Lin et al., 2016). In many of these tissues the canonical mediators of Reelin signal transduction are not expressed, whereas interactions of hitherto unknown significance, such as low-affinity binding to the LDL receptor (D’Arcangelo et al., 1999), might turn out to be of physiological significance and hint to as yet unknown or poorly defined functions of Reelin that might be unrelated to signaling, e.g., sequestering of coagulation factors (Tseng et al., 2014).

## Author Contributions

HHB and PM: designed, wrote and approved the review.

## Funding

Work in the laboratory of HHB and PM is supported by the Bundesministerium für Bildung und Forschung (BMBF) (ReelinSys, 0316174C) and the Deutsche Forschungsgemeinschaft (DFG) (SFB974/B10).

## Conflict of Interest Statement

The authors declare that the research was conducted in the absence of any commercial or financial relationships that could be construed as a potential conflict of interest.
